# (R)-(+)-β-Citronellol and (S)-(−)-β-Citronellol in Combination with Amphotericin B against Candida Spp.

**DOI:** 10.3390/ijms21051785

**Published:** 2020-03-05

**Authors:** Daniele Silva, Hermes Diniz-Neto, Laísa Cordeiro, Maria Silva-Neta, Shellygton Silva, Francisco Andrade-Júnior, Maria Leite, Jefferson Nóbrega, Maria Morais, Juliana Souza, Lyvia Rosa, Thamara Melo, Helivaldo Souza, Aleson Sousa, Gregório Rodrigues, Abrahão Oliveira-Filho, Edeltrudes Lima

**Affiliations:** 1 Postgraduate Program in Bioactive Natural and Synthetic Products, Federal University of Paraíba, João Pessoa 58051-970, Brazil; hermes.dn@hotmail.com (H.D.-N.); laisavilar@gmail.com (L.C.); neves_neta@hotmail.com (M.S.-N.); shellygton@hotmail.com (S.S.); juniorfarmacia.ufcg@outlook.com (F.A.-J.); denisecaiana@yahoo.com.br (M.L.); jeffersonrodriguesn@hotmail.com (J.N.); frannciellysimoes@gmail.com (M.M.); lyvinhasr14@hotmail.com (L.R.); th.rmelo@ltf.ufpb.br (T.M.); edelolima@yahoo.com.br (E.L.); 2 Department of Chemistry, Federal University of Paraíba, João Pessoa 58051-970, Brazil; julianakelly71@gmail.com (J.S.); helivaldog3@gmail.com (H.S.); 3 Postgraduate Program in Drug Development and Technological Innovation, Federal University of Paraíba, João Pessoa 58051-970, Brazil; aleson_155@hotmail.com; 4 Postgraduate Program in Dentistry, Federal University of Paraíba, João Pessoa 58051-970, Brazil; gregorio_marcio1@yahoo.com.br; 5 Health and Rural Technology Center, Federal University of Campina Grande, Patos 58700-970, Brazil; abrahao.farm@gmail.com

**Keywords:** isomers, (R)-(+)-β-citronellol, (S)-(−)-β-citronellol, *candida* spp., antifungal activity

## Abstract

The enantiomers (R)-(+)-β-citronellol and (S)-(−)-β-citronellol are present in many medicinal plants, but little is understood about their bioactivity against *Candida* yeasts. This study aimed to evaluate the behavior of positive and negative enantiomers of β-citronellol on strains of *Candida albicans* and *C. tropicalis* involved in candidemia. The minimum inhibitory concentration (MIC) and minimum fungicide concentration (MFC) were determined. The evaluation of growth kinetics, mechanism of action, and association studies with Amphotericin B (AB) using the checkerboard method was also performed. R-(+)-β-citronellol and S-(−)-β-citronellol presented a MIC_50%_ of 64 µg/mL and a MFC_50%_ of 256 µg/mL for *C. albicans* strains. For *C. tropicalis*, the isomers exhibited a MIC_50%_ of 256 µg/mL and a MFC_50%_ of 1024 µg/mL. In the mechanism of action assay, both substances displayed an effect on the fungal membrane but not on the fungal cell wall. Synergism and indifference were observed in the association of R-(+)-β-citronellol and AB, while the association between S-(−)-β-citronellol and AB displayed synergism, additivity, and indifference. In conclusion, both isomers of β-citronellol presented a similar profile of antifungal activity. Hence, they can be contemplated in the development of new antifungal drugs providing that further research is conducted about their pharmacology and toxicity.

## 1. Introduction

Optical isomers, or enantiomers, are non-overlapping images which possess the same physicochemical properties, except in the way they deviate polarized light and odor. Further divergent behavior can be revealed when they are in biological environments [1].

The occurrence of chirality in natural products is very common, and efforts to understand their biological profiles and origins in biosynthetic pathways are constant [2]. These products present considerable importance to the pharmaceutical industry due to their ability to serve as active molecules or as prototypes for obtaining specific pharmacological and toxicological profiles, which often cannot be obtained without more expensive chemical synthesis [3].

Terpenes represent one of the larger classes of secondary metabolites, comprising more than 30,000 compounds. Due to their diversity and biological activity, these molecules often make enormous contributions. Six classes of medications, namely taxanes, steroids, tocopherols, ingenanes, artemisinins, and cannabinoids, owe their existence to molecules of this class, revealing how much this group has contributed in the last century to both modern medicine, and many different industrial segments [4,5,6,7,8].

Enantiomers are generally present in terpenoids, yet they are restricted to certain subclasses, namely the monoterpene, sesquiterpenes, diterpenes (in a lesser proportion), and sesterterpenes (with a single case identified) [8]. β-citronellol is an enantiomer found in the monoterpene class [9], and although its optically active forms are present in the composition of essential oils of different species of medicinal plants in Central America, South America, Asia, and Africa, its isomeric optical forms are still poorly explored [1,10,11,12]. For example, it was found that the R-(+) isomer of β-citronellol has anticonvulsant activity and this effect is attributed to its ability to reduce neuronal excitability through the blocking of voltage-dependent Na+ channels in rodents [13]. Other pharmacological behaviors are described but involving only the racemic mixture, such as the inhibition of a number of factors involved in the processes that lead to inflammation. This was studied in macrophages RAW2647 [14], vasodilator action [15], and inhibitors of muscle contraction [16].

Due to negligence, nosocomial fungal infections with high morbidity and mortality rates have greatly increased in both developed and developing countries [17,18]. Thus, over more than two decades, *Candida* spp. blood infections have become a growing problem [19]. In Brazil, the incidence of candidemia is around 249 cases per 100,000 hospitalized patients [20], with *C. albicans* as the principal agent responsible (globally as well) and with *C. tropicalis* and *C. parapsilosis* closely following [21]. Although drug interventions to treat these infections exist, *Candida spp.* resistance is a persistent problem. Certain species are intrinsically resistant to many antifungal agents, such as *C. glabrata* and *C. krusei* against fluconazole, and *C. lusitaniae* against AB. In other *Candida* species, antifungal resistance often develops over time as a result of incorrect therapeutic management [22,23,24].

Given the need for new antifungal agents against *Candida*, the lack of information on the bioactive properties of β-citronellol isomers, and the importance of studying enantiomers in biological environments to obtain new drugs, the biological activity of (R)-(+)-β-citronellol and (S)-( −)-β-citronellol enantiomers against clinical isolates of *Candida* spp. obtained from candidemia was chosen for study. We assessed the nature of their activity, their mechanisms of action, and their behavior in association with the antifungal AB.

## 2. Results

### 2.1. Determination of MIC and MFC of (R)-(+)-β-Citronellol, (S)-(−)-β-Citronellol, and AB on C. Albicans and C. Tropicalis

Determinations of MIC and MFC for R-(+)-β-citronellol against strains of *C. albicans* and *C. tropicalis* are expressed in Table 1.

The MIC_50%_ (minimum inhibitory concentration capable of inhibiting 50% of the fungal strain) found for R-(+)-β-citronellol was 64 µg/mL and its MFC_50%_ (minimum fungicide concentration capable of killing 50% of the fungi strain) was 256 µg/mL against strains of *C. albicans*. The MIC_50%_ of *C. tropicalis* was 256 µg/mL and its MFC_50%_: was 1024µg/mL (Table 1).

Determination of the MIC and MFC for S-(−)-β-citronellol against strains of *C. albicans* and *C. tropicalis* are expressed in Table 2.

Similar to what was evidenced by R-(+)-β-citronellol against *C. albicans* yeasts, S-(−)-β-citronellol also presented an MIC_50%_ of 64 µg/mL and an MFC_50%_: of 256 µg/mL against these strains. For *C. tropicalis*, the mono-terpenoid obtained an MIC_50%_: of 128 µg/mL and an MFC_50%_ of 512 µg/mL (Table 2).

When comparing the MIC values of (R)-(+)-β-citronellol and (S)-(−)-β-citronellol (Table 1 and Table 2), it was observed that none of the phytoconstituents presented activity higher than the other against the strains of *C. albicans* (*p* = 0.505) and *C. tropicalis* (*p* = 0.485). The same was observed when comparing the MFC of these phytoconstituents for the *Candida albicans* (*p* = 0.878) and non-*albicans* species (*p* = 0.310).

Regarding the sensitivity profile to AB, this polyene was effective for most of the tested yeasts with MICs values equal or lower than 1 μg/mL (MICs above this concentration in strains of *Candida* spp. configures resistance to this antifungal drug), except for two clinical isolates of *C. albicans*: *C. a.* LM-612, with an MIC of 8 μg/mL; and *C. a.* LM-852, with an MIC of 32 μg/mL.

### 2.2. Effect of (R)-(+)-β-Citronellol and (S)-(−)-β-Citronellol on Yeast Growth Kinetics

**Figure 1:**
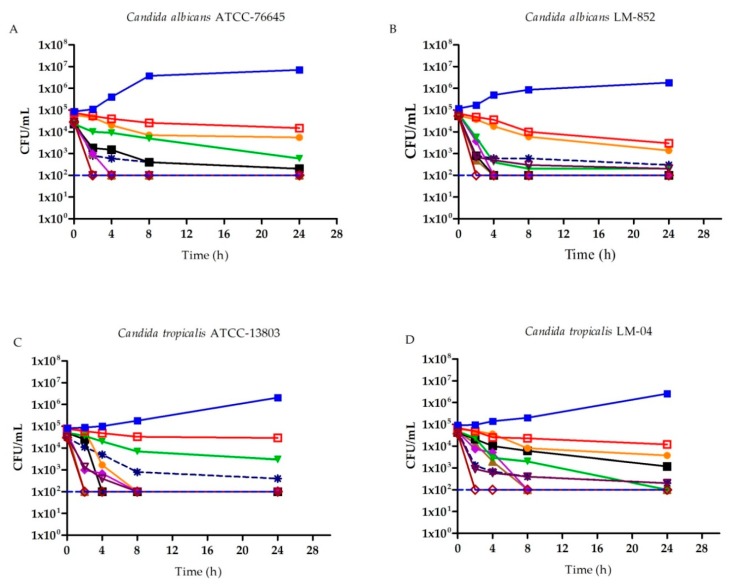
Microbial death curve (Log_10_ CFU/mL) for strains of *C. albicans* and *C. tropicalis*, under different concentrations (MIC, MICx2, MICx4) of AB, (R)-(+)-β-citronellol, and (S)-(−)-β-citronellol in different time intervals. (A) Microbial death curve for *C. albicans* ATCC 76645. (B) Microbial death curve for *C. albicans* LM-852. (C) Microbial death curve for *C. tropicalis* ATCC 13803. (D) Microbial death curve for *C. tropicalis* LM-04. (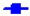) Control of viability for strains (*C. albicans* ATCC 76645, *C. albicans* LM-852, *C. tropicalis* ATCC 13803, and *C. tropicalis* LM-04); (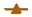) (S)-(−)-β-citronellol MICx4; (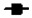) (S)-(−)-β-citronellol MICx2; (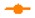) (S)-(−)-β-citronellol MIC; (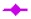) (R)-(+)-β-citronellol MICx4; (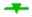) (R)-(+)-β-citronellol MICx2; (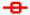) (R)-(+)-β-citronellol MIC; (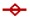) AB MICx4; (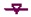) AB MICx2; (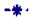) AB MIC; (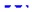) Detection limit.

Fungal growth was analyzed based on the microbial death curve of *C. albicans* and *C. tropicalis* strains using different concentrations of (R)-(+)-β-citronellol, (S)-(−)-β-citronellol, and AB (a standard antifungal) as a function of time (Figure 1). In this essay, the number of colony-forming units (CFU) is counted in order to determine if the product exerts fungicidal or fungistatic activity. This also allows for the establishment of a dynamic relationship between concentration and activity throughout the analyzed time intervals.

In Figure 1A we see that, for the *C. albicans* ATCC-76645 lineage treated with MICx2 (S)-(−)-β-citronellol, there is a reduction of more than 2 lg CFU/mL at 8 h of assay, which falls to 3 lg CFU/mL when treated with MICx4 at 4 h. This same behavior was observed for (R)-(+)-β-citronellol. However, the reduction of 2 lg CFU/mL occurred only at 24 h. For the *C. albicans* LM-852 clinical isolate, the decrease consisted of 3 log_10_ CFU/mL at 4 h of test with MICx4 for the positive β-citronellol isomer, and MICx2 for the negative isomer in Figure 1B.

For the *C. tropicalis* ATCC-13803 strain, (R)-(+)-β-citronellol at MICx4, promotes 3 lg CFU/mL reduction at 8 h of assay. (S)-(−)-β-citronellol at MICx4 promotes the same effect at a lower concentration of MICx2, Figure 1C. A decrease of 3 lg CFU/mL is seen at 24 h for *C. tropicalis* LM-04 when treated with (R)-(+)-β-citronellol at MICx2, and when treated with (S)-(−)-β-citronellol at MICx4, at 8 h, Figure 1D.

### 2.3. Mechanism of Action: (R)-(+)-β-Citronellol and (S)-(−)-β-Citronellol

Sorbitol assays performed in this study demonstrated that neither (R)-(+)-β-citronellol nor (S)-( −)-β-citronellol exert an antifungal effect through interaction with the cell wall. This is observed by the MIC of the substances, which remained unchanged in the presence and absence of sorbitol (osmotic protector) (Table 3).

In the ergosterol tests, for the strains of *C. albicans* (ATCC-76645 and LM-852) and *C. tropicalis* (*ATCC*-13803 and LM-04) it was observed that, for the positive isomer of β-citronellol, when in the presence of exogenous ergosterol, the MIC increases 128 times. For its negative isomer, an increase of 128 times of the MIC in the presence of this sterol was also observed against almost all specimens of *Candida* spp., except for *C. tropicalis* LM-04 where the increase was 64 times (Table 4).

### 2.4. Interaction Profile of R-(+)-β-Citronellol and S-(−)-β-Citronellol with AB

The results of the association assays of both R-(+)-β-citronellol and S-(−)-β-citronellol with AB against the tested strains are displayed in Table 5. In the combination of R-(+) β-citronellol and AB, two effects were observed: Synergism for *C. albicans* LM-612 (FICI: 0.1875) and *C. albicans* LM-852 (FICI: 0.1875), and indifference for *C. albicans* ATCC 76645 (FICI: 1.0625), *C. tropicalis* ATCC 13803 (FICI: 1.0625), and *C. tropicalis* LM-04 (FICI: 1.025).

For S-(−)-β-citronellol with AB, three distinct effects were seen: additivity for *C. albicans* ATCC-76645 (FICI: 0.5625), synergism for *C. albicans* LM-612 (FICI: 0.1875) and *C. albicans* LM-852 (FICI: 0.3125), and indifference for the other strains of *C. tropicalis:* ATCC 13803 (FICI: 1.0625) and LM-04 (FICI: 2). When performing the statistical analysis about the FICI of the association between (R)-(+)-β-citronellol and (S)-(−)-β-citronellol associated with AB, to analyze which was most promising, no statistically significant differences were observed between them against the strains of *C. albicans* (*p* = 1) and *C. tropicalis* (*p* = 1).

## 3. Discussion

Most studies involving the analysis of β-citronellol anti-*Candida* spp. activity involve racemic mixtures [25,26]. In the only study available involving evaluation of antimicrobial activity of the (R)-(+)-β-citronellol and (S)-(−)-β-citronellol isomers in the literature for strains of *C. albicans* (*C. albicans* ATCC-10231 and *C. albicans* ATCC-24433), the positive isomer presented respective MIC’s of 120 and 1000 μg/mL. For *C. tropicalis* (*C. tropicalis* ATCC-1369, and *C. tropicalis* ATCC-750) the MIC’s were 2000 μg/mL. For the negative isomer, the MIC’s obtained against the *C. albicans* strains were respectively 60 and 1000 μg/mL, and for *C. tropicalis* they were 1000 and 500 μg/mL [27]. The findings of the present study, combined with these results, reveal that both isomers are strongly active against *Candida* spp. when considering the MICs _50%_ obtained [28,29].

Although the MFC_50%_ revealed for (R)-(+)-β-citronellol and (S)-(−)-β-citronellol diverged between the *Candida* strains, the activity evidenced by both isomers is putatively fungicidal. The MFC_50%_/MIC_50%_ ratio was thus equal to 4 (this value, which characterizes effects as fungicidal and as non-fungistatic occurs when the MFC_50%_/MIC_50%_ ratio > 4) [30]. A profile of this kind was also revealed in racemic mixtures against *Candida albicans* isolates from different anatomical sites [26].

Regarding cases of resistance to AB, two other non-*albicans* strains also derived from invasive candidiasis were resistant to this polyene [31]. In light of the current information, the molecular mechanisms of resistance to this antifungal drug, which are strictly related to the failure in treatment of these invasive fungal infections, are highlighted [32,33]. According to the literature, some of these mechanisms include defects in the genes responsible for the biosynthesis of ergosterol such as ERG1, ERG2, ERG3, ERG4, ERG6 and ERG11, protective mechanisms developed against oxidative damage caused by polyenes, changes in the sterol/phospholipid ratio, as well as reorganization or alteration of ergosterol in the cellular membrane [34,35].

As for death kinetics, we observed that the rate and extent of antifungal activity varied between the four *Candida* spp. strains for both (R)-(+)-β-citronellol and (S)-(−)-β-citronellol. The behavior of the positive and negative isomers of β-citronellol coincided with the MFC assay findings, and the antifungal activity improved with higher concentrations. Thus, the higher the concentrations of these phytoconstituents, the shorter the time necessary to achieve fungicidal effect.

This same profile was observed in time-to-kill assays performed using several concentrations of *Cymbopogon winterianus* essential oil (citronellol is a principal compound) against two *C. albicans* yeasts [36].

Studies of microorganism death kinetics were not observed in the literature for these β-citronellol isomers. Hence, this is the first time this type of experiment has been carried out for these compounds.

The mechanism by which monoterpenes act against microorganisms is not yet fully understood. However, it has been reported that these natural products cause injury to the plasmatic membrane by inducing alterations in fluidity and permeability, generating disturbances in structural functionality [37].

In the present study, the isomers of β-citronellol were demonstrated as acting against the *Candida* ssp. cytoplasmic membrane. The considerable increase in MIC in the presence of exogenous ergosterol, reveals that it forms a protective barrier, preventing direct interaction between the phytoconstituents and the ergosterol present in the fungal membranes of the *C. albicans* and *C. tropicalis* strains used in this assay.

It is also noteworthy that these monoterpenes act differently against filamentous fungi since of the possible mechanisms of action for citronellol against *Trichophyton rubrum* ATCC-1683, where this phytoconstituents in the racemic mixture exhibited action upon the fungal cell wall and by inhibiting the ergosterol biosynthesis [38].

The intimate layers (basal layers) of most fungi consist of β-(1.3) glucan, β-(1.6) glucan, and chitin, but components of the external layers differ substantially. Therefore, divergences in monoterpene activity against the fungi are possibly related to variations in cytoskeletal system configurations between yeast and filamentous cells, and other physiological aspects which have not yet been fully clarified [39,40,41].

Antimicrobial combinations can result in synergism, antagonism, indifference, or additivity, and using the *checkerboard* method it is possible to evaluate these interactions against different pathogens [42,43,44].

The association between natural products and antifungal agents is employed as a feasible approach to bypass the limitations of antifungal monotherapy [44,45,46], seeking to broaden the spectrum of therapeutic efficacy, prevent the development of antifungal resistance, and reduce toxicity. Thus, this strategy seeks to achieve greater success in treatment, which has already been observed in combined therapies of certain antifungals [46,47,48].

In this study, although indifference was visualized for associations against certain fungal isolates, the presence of synergistic effect against *C. albicans* LM-612 and *C. albicans* LM-852 (both resistant to the antifungal standard) was obtained by combining the (R,S) β-citronellol isomers with AB. Additivity against *C. albicans* ATCC 76645 for the S-(−)-β-citronellol–AB association made it possible to infer that in the future, the isomers can be used in antifungal drug associative therapies. In a previous association study conducted by Silva et al. [26], a profile of synergism dominated for (±)-β-citronellol and the three azolic antifungal agents (fluconazole, miconazole, and itraconazole) when used against *C. albicans* yeasts. This included strains that were previously resistant to these antifungal agents, and suggested resistance reversal in these strains. This was also be observed in our research.

## 4. Materials and Methods

### 4.1. Phytoconstituents

(R)-(+)-β-citronellol and (S)-(−)-β-citronellol (Sigma-Aldrich^®^, São Paulo, Brazil) were solubilized in 10% dimethyl sulfoxide (DMSO) and 2% Tween 80 in a test tube, and supplemented with sterilized distilled water to finally obtain an emulsion in the concentration of 2048μg/mL [49].

### 4.2. Culture Media

To maintain the strains and to perform the antifungal activity assays, Sabouraud Dextrose Agar (SDA) (Difco Laboratories Ltd., Sparks, MD, USA) and Roswell Park Memorial Institute (RPMI) medium, with L-glutamine and without bicarbonate (respectively) were used (INLAB, São Paulo, SP, Brazil), and prepared according to the descriptions of the manufacturers.

### 4.3. Microorganisms and Inoculum Preparation

In this study, 8 strains of *C. albicans* (ATCC-76645, LM-52, LM-80, LM-92, LM-240, LM-271, LM-612, and LM-852) and 6 strains of *C. tropicalis* (ATCC-13803, LM-01, LM-04, LM-06, LM-12, and LM-18) were used, totaling 14 strains. Further, two strains originated from the American Type Culture Collection (ATCC) and 12 strains were of clinical origin. All of the fungal strains was obtained from the collection of Dr. Thompson Lopes de Oliveira, of the laboratory for research in antibacterial and antifungal activity of natural and/or bioactive products of the Department of Pharmaceutical Sciences, Health Sciences Center, Federal University of Paraíba, and were registered in the National System of Genetic Heritage Management and Associated Traditional Knowledge under the numbers: A2DA181 (*C. albicans* strains) and AD662FE (*C. tropicalis* strains). The strains were maintained in SDA at a temperature of 4 °C. The inoculum was prepared from cultures of *C. albicans* and *C. tropicalis* in SDA at 35 ± 2 °C for 24–48 h. Colonies of these yeasts were suspended in a sterile solution of NaCl (0.9%), agitated in a vortex apparatus, and adjusted according to the 0.5 McFarland standard in order to obtain an inoculum of 1–5 × 10^6^ CFU/mL, which was employed in the further studies of this research.

### 4.4. Determination of MIC

The antifungal activity assays were performed according to the protocols of Cleeland and Squires (1991) [50], Hadacek and Greger (2000) [51], and CLSI (2008) [52]. Determination of the MIC’s for (R)-(+)-β-citronellol and (S)-(−)-β-citronellol, on the *Candida* strains was performed using broth microdilution technique in a 96-well microplate for cell culture (INLAB, São Paulo, Brazil) with a “U”-shaped bottom. Initially, 100 μL of double-concentrated RPMI-1640 was distributed to the wells of the microdilution plates. Then, 100 μL of emulsion of each test product (also double-concentrated) was dispensed to the wells of the first line of the plate. By means of serial dilution at a ratio of two, concentrations of 1024 μg/mL to 2 μg/mL were obtained. Finally, 10 μL of the *Candida* inoculum was added to the wells, where each column of the microplate referred to a specific fungal lineage.

MIC determination was also performed for AB (the standard antifungal agent) with a control of fungal viability (wells containing 100 μL of culture media broth and 10 µL of fungal inoculum resulting in1–5 × 10^5^ CFU/mL in each well). Plates were incubated at 35 ± 2 °C for 24–48 h. The MIC was defined as the lowest concentration of the product capable of producing the visible inhibition of fungal growth (observed in the wells) as compared to the controls. The result was expressed as the arithmetic mean of the MIC obtained, performed in triplicate.

The antifungal activity of (R)-(+)-β-citronellol and (S)-(−)-β-citronellol was interpreted and considered either active or inactive, according to the following criteria: 50–500 μg/mL = strong/optimum activity; 600–1500 μg/mL = moderate activity; > above 1500 μg/mL = weak activity or inactive [28,29].

In order to detect AB resistant strains of *Candida* spp., the protocols of the document M27-A2 by CLSI were employed, where MIC > 1μg/mL accounts for resistance and MIC ≤ 1μg/mL means susceptibility [53].

### 4.5. Determination of MFC

Briefly, 10 μL aliquots of supernatant from the wells where complete inhibition of fungal growth had occurred (MIC, MICx2, and MICx4) were sub-cultured in 100 μL of RPMI-1640, in new cell culture plates. The newly prepared plates were then incubated at 35 ± 2 °C for 24–48 h. MFC was considered as the lowest concentration in which there was no visible fungal growth in the well. The assays were performed in triplicate and the results expressed as the arithmetic mean of MFC’s obtained in the three tests [54,55].

### 4.6. Effect of the Test Products on the Yeasts’ Growth Kinetics

The study for (R)-(+)-β-citronellol and (S)-(−)-β-citronellol ’s effect on the growth curve of the fungal strains was performed using the methodology described by Klepser et al. [56] with some modifications.

Two strains of *C. albicans* (ATCC 76645 and LM-852) and two strains of *C. tropicalis* (ATCC 13803 and LM-04) were used to perform yeast growth kinetics. In this assay, the influence of minimal inhibitory concentrations of (R)-(+)-β-citronellol, (S)-(−)-β-citronellol, and AB (the standard control drug) on the growth curves of the selected yeasts was assessed over a period of 24 h.

Initially, 100 µL of RPMI-1640, was added to a “U”-shaped bottom, 96-well microplate. Subsequently, 10 µL of the supernatant of the wells corresponding to the inhibitory concentration and two mediums with higher concentrations (MIC, MICx2 and MICx4) were added to the wells and incubated for a period of 24–48 h at 35 ± 2 °C.

Subsequently, a 10 µL aliquot of each concentration was collected with calibrated bacteriological loops (INLAB, São Paulo, Brazil) and streaked uniformly in the shape of stretched marks on the SDA surface in 90 × 15mm Petri dishes (INLAB, São Paulo, Brazil), using time intervals of 0, 2, 4, 8, and 24 h. At each interval, fungal viability control plates were also prepared. The inoculated plates were incubated at 35 ± 2 °C for 48 h.

The experiment was carried out in triplicate and the curves were constructed by plotting the average colony count (log_10_ CFU/mL) as a function of time (hours). The fungicidal effectiveness of the drug was considered when there was a reduction in microbial growth of greater than or equal to 3 log_10_ (≥ 99.9%) from the initial inoculum. Fungistatic activity was considered when there was a reduction in microbial growth of lower than 3 log_10_ (<99,9%) [56].

### 4.7. Fungal Cell Wall Effect (Sorbitol Assay)

Determination of the MICs of (R)-(+)-β-citronellol and (S)-(−)-β-citronellol in the presence of sorbitol 0.8 M (an osmotic protector stabilizing fungi protoplasts) was performed using the microdilution technique in cell culture plates containing 96 wells with a “U”-shaped bottom, and in triplicate, similar to Section 4.4. To each well of the plate was added 100 µL of RPMI-1640, supplemented with sorbitol molecular weight 182.17g (Vetec QuímicaFina LTDA–Rio de Janeiro, RJ, Brazil), with both double-concentrated. Subsequently, 100 µL of the test product emulsion (also double-concentrated) was dispensed to the wells of the first line of the plate. Using serial dilution at a ratio of two, the needed concentrations of the products were obtained in each well with a final sorbitol concentration of 0.8 M. Finally, 10 µL of the inoculum of the *C. albicans* (ATCC 76645 and LM-852) and *C. tropicalis* (ATCC-13803 and LM-04) strains was added to the wells, where each column of the plate referred to a specific fungal strain.

A microorganism control was performed by injecting the culture medium with 200 µL of sorbitol (0.8 M) and 10 µL of the inoculum of each species into each well. A sterility control was also performed where 200 µL of the culture medium was inserted into a well without fungal inoculum. The plates were aseptically closed and incubated at 35 ± 2 °C for 24–48 h until the time of reading [57].

### 4.8. Interaction with Fungal Cell Membrane Ergosterol (Ergosterol Assay)

To determine whether the β-citronellol enantiomers bind to fungal membrane sterols, the MIC of these products for *Candida* spp. was determined with and without the addition of ergosterol. If the activity of the product is caused by the binding to ergosterol, exogenous ergosterol will prevent binding to fungal membrane ergosterol, and as a consequence the MIC of this product will increase in relation to the assay control. If the MIC of the product remains unchanged in the presence of different exogenous concentrations of ergosterol, it is suggested that the compound does not act through binding to membrane ergosterol. Similarly, it can be observed whether the behavior is specific to ergosterol.

Determination of the MIC’s for (R)-(+)-β-citronellol and (S)-(−)-β-citronellol against strains of *C. albicans* (ATCC 76645 and LM-852) and *C. tropicalis* (ATCC 13803 and LM-04) was performed through microdilution using culture plates with 96 wells and a “U” shaped bottom in triplicate, similar to the protocol given in Section 4.4. RPMI-1640 was used with and without the addition of ergosterol at 400 µg/mL (Sigma-Aldrich^®^, São Paulo, Brazil). A microorganism control was performed using 100 µL of the culture medium and ergosterol in equal concentrations, and 10 µL of each species inoculum. The plates were sealed and incubated at 35 ± 2 °C for 24–48 h and reading was subsequently performed [58].

### 4.9. Study of β-Citronellol Associations with AB – Checkerboard Method

The checkerboard method was employed to assess the effects of the association between the test products and a standard antifungal drug: (R)-(+)-β-citronellol with AB, and (S)-(−)-β-citronellol with AB against strains of *C. albicans* (ATCC-76645, LM-852, and LM-612) and *C. tropicalis* (ATCC-13803 and LM-04). Initially, 100 μL of RPMI-1640 was added to the wells of a sterile microplate containing 96 wells with a “U” shaped bottom. At the same time, dilutions of each of the isomers of β-citronellol and AB were prepared in tubes so that concentrations higher than MIC as well as subinhibitory were obtained (MICx8, MICx4, MICx2, MIC, MIC÷2, MIC÷4, MIC÷8, and MIC÷16). Then, 50 μL of each isomer in the various concentrations was added to 50 μL of AB in each concentration in the microplate. In order to obtain different combinations between the concentrations, the substances were added in different directions in the microplate (isomers added horizontally, and AB added vertically). Finally, 20 μL of the fungal suspension was added in all the wells. The assay was performed in triplicate, and the microplates were incubated for 24–48 h at 35 ± 2 °C [42,59].

After incubation, the fractional inhibitory concentration index (FICI) was calculated using the following equation: FICI = FIC_A_ + FIC_B_, where ‘A’ represents the test product and ‘B’ the standard antifungal. The FIC_A_ is calculated by the combined MIC_A_/isolated MIC_A_ ratio, while the FIC_B_ is calculated by the combined MIC_B_/isolated MIC_B_ ratio. The index is interpreted as follows: synergism (FICI ≤ 0.5), additivity (0.5 < FICI < 1.0), indifference (1 < FICI < 4), and antagonism (FICI ≥ 4,0) [59,60].

### 4.10. Statistical Analysis

Statistical analysis was performed using the Mann–Whitney U test between the MIC and MFC values of (R)-(+)-β-citronellol and (S)-(−)-β-citronellol for *C. albicans* and *C. tropicalis* strains. In addition, the FICI values for both phytoconstituents associated with AB were compared. The results were considered statistically significant when *p* < 0.05 for the rejection of the null hypothesis. The Statistical Package for The Social Sciences software, version 13.0, was used for these analyses.

The data from the assays of the microbial growth kinetics curve was plotted as log_10_ CFU/mL according to the time intervals and the concentrations of the substances studied. For this plotting, GraphPad Prism (version 6.0 for Windows, San DIEGO, CA-EUA) software was used. The analysis parameter to verify the presence of differences between the control curve and test curves were expressed through changes in the CFU/mL values.

## 5. Conclusions

In accordance with the results obtained in this in vitro research, strong antifungal activity (being fungicidal) is displayed by (R)-(+)-β-citronellol and (S)-(−)-β-citronellol without a statistical difference between them. The isomers activity is caused by damage to the *Candida* spp. cell membrane. The association results with AB demonstrated differing effects depending on the fungal strain employed. The synergism and additivity presented suggest that these monoterpenes may well be used for reducing antifungal resistance. Provided that more studies are carried out to better understand their bioactivity and toxicity profiles, these isomers that present clear antifungal activity may well improve the development of new antifungal therapeutic alternatives.

## Figures and Tables

**Table 1:** MIC and MFC for R-(+)-β-citronellol and AB against strains of *C. albicans* and *C. tropicalis*.

Strains	R-(+)-β-Citronellol		AB		Control *
	MIC (µg/mL)	MFC (µg/mL)	MIC (µg/mL)	MFC (µg/mL)	
*C. albicans* ATCC 76645	64	256	0.125	0.25	+
*C. albicans* LM-52	64	128	0.125	0.25	+
*C. albicans* LM-80	256	512	1	2	+
*C. albicans* LM-92	64	64	0.125	0.125	+
*C. albicans* LM-240	64	512	0.5	2	+
*C. albicans* LM-271	64	128	0.125	0.5	+
*C. albicans* LM-612	256	1024	8	8	+
*C. albicans* LM-852	256	1024	32	128	+
*C. tropicalis* ATCC-13803	64	256	0.5	0.5	+
*C. tropicalis* LM-01	256	1024	1	2	+
*C. tropicalis* LM-04	128	256	0.125	0.5	+
*C. tropicalis* LM-06	256	1024	1	4	+
*C. tropicalis* LM-12	256	1024	1	4	+
*C. tropicalis* LM-18	512	1024	1	4	+

MIC: Minimum inhibitory concentration; MFC: minimum fungicidal concentration; * Control of microorganism growth in RPMI-1640.

**Table 2:** MIC and MFC for S-(−)-β-citronellol and AB against strains of *C. albicans* and *C. tropicalis*.

Strains	S-(−)-β-Citronellol		AB		Control *
	MIC (µg/mL)	MFC (µg/mL)	MIC (µg/mL)	MFC (µg/mL)	
*C. albicans* ATCC-76645	32	128	0.125	0.25	+
*C. albicans* LM-52	32	128	0.125	0.25	+
*C. albicans* LM-80	128	1024	1	2	+
*C. albicans* LM-92	64	512	0.125	0.125	+
*C. albicans* LM-240	128	256	0.5	2	+
*C. albicans* LM-271	64	256	0.125	0.5	+
*C. albicans* LM-612	128	1024	8	8	+
*C. albicans* LM-852	128	256	32	128	+
*C. tropicalis* ATCC 13803	64	128	0.5	0.5	+
*C. tropicalis* LM-01	128	512	1	2	+
*C. tropicalis* LM-04	64	256	0.125	0.5	+
*C. tropicalis* LM-06	256	512	1	4	+
*C. tropicalis* LM-12	256	512	1	4	+
*C. tropicalis* LM-18	256	1024	1	4	+

MIC: Minimum inhibitory concentration; MFC: minimum fungicidal concentration; * Control of microorganism growth in RPMI-1640.

**Table 3:** Effect of R-(+)-β-citronellol and S-(−)-β-citronellol on the strains of *C. albicans* (ATCC-76645 and LM-852) and *C. tropicalis* (ATCC-13803 and LM-04) in the absence and presence of sorbitol 0.8 M.

Strains	R-(+)β-citronellol		S-(−)β-citronellol	
	MIC (µg/mL)		MIC (µg/mL)	
	Absence of Sorbitol	Presence of Sorbitol	Absence of Sorbitol	Presence of Sorbitol
*C. albicans* ATCC 76645	64	64	32	32
*C. albicans* LM-852	256	256	128	128
*C. tropicalis* ATCC13803	64	64	64	64
*C. tropicalis* LM-04	128	128	64	64

MIC: Minimum inhibitory concentration.

**Table 4:** Effect of R-(+)-β-citronellol and S-(−)-β-citronellol on the tested strains of *C. albicans* (ATCC-76645 and LM-852) and *C. tropicalis* (ATCC-13803 and LM-04) in the absence and presence of ergosterol 400 µg/mL.

Strains	R-(+)β-citronellol		S-(−)β-citronellol	
	MIC (µg/mL)		MIC (µg/mL)	
	Absence of Ergosterol	Presence of Ergosterol	Absence of Ergosterol	Presence of Ergosterol
*C. albicans* ATCC-76645	64	8192	32	4096
*C. albicans* LM-852	256	32768	128	16384
*C. tropicalis* ATCC-13803	64	8192	64	8192
*C. tropicalis* LM-04	128	16384	64	4096

MIC: Minimum inhibitory concentration.

**Table 5:** Determination of the fractional inhibitory concentration index (FICI) of the association of R-(+)-β-citronellol, S-(−)-β-citronellol and AB on strains of *C. albicans* and *C. tropicalis*.

Strains	FIC_A_	FIC_B_	FICI	Interaction Type	FIC_A_	FIC_B_	FICI	Interaction Type
	R-(+)-β-Citronellol	AB			S-(−)-β-Citronellol	AB		
*C. albicans* ATCC 76645	0.0625	1	1.0625	Indifferent	0.0625	0.5	0.5625	Additivity
*C. albicans* LM-612	0.0625	0.125	0.1875	Synergism	0.125	0.0625	0.1875	Synergism
*C. albicans* LM-852	0.0625	0.125	0.1875	Synergism	0.0625	0.25	0.3125	Synergism
*C. tropicalis* ATCC 13803	0.0625	1	1.0625	Indifferent	0.0625	1	1.0625	Indifferent
*C. tropicalis* LM-04	0.0625	1	1.025	Indifferent	1	1	2	Indifferent

FIC_A_: fractioned inhibitory concentration of “A”, represents (R-(+)-β-citronellol/S-(−)-β-citronellol); FIC_B_: fractioned inhibitory concentration of “B” represents the standard antifungal (AB). FICI: fractionated inhibitory concentration index, obtained by the sum of FIC_A_ and FIC_B_, characterizes the type of interaction (synergism, additivity, indifference, or antagonism).

## Abbreviations

DMSO	Dimethyl Sulfoxide
SDA	Sabouraud Dextrose Agar
RPMI	Roswell Park Memorial Institute
MIC	Minimum Inhibitory Concentration
ATCC	American Type Culture Collection
MFC	Minimum Fungicide Concentration
AB	Amphotericin B
CFU	Colony Forming Units
FICI	Fractional Inhibitory Concentration Index
